# Political Attitudes and Disease Threat: Regional Pathogen Stress Is Associated With Conservative Ideology Only for Older Individuals

**DOI:** 10.1177/01461672231183199

**Published:** 2023-07-10

**Authors:** Gordon D. A. Brown, Lukasz Walasek, Timothy L. Mullett, Edika G. Quispe-Torreblanca, Corey L. Fincher, Michal Kosinski, David Stillwell

**Affiliations:** 1 University of Warwick, Coventry, UK; 2 University of Oxford, UK; 3 Stanford Graduate School of Business, CA, USA; 4 University of Cambridge, UK

**Keywords:** pathogen stress, infection, ideology, conservatism

## Abstract

What environmental factors are associated with individual differences in political ideology, and do such associations change over time? We examine whether reductions in pathogen prevalence in U.S. states over the past 60 years are associated with reduced associations between parasite stress and conservatism. We report a positive association between infection levels and conservative ideology in the United States during the 1960s and 1970s. However, this correlation reduces from the 1980s onwards. These results suggest that the ecological influence of infectious diseases may be larger for older people who grew up (or whose parents grew up) during earlier time periods. We test this hypothesis by analyzing the political affiliation of 45,000 Facebook users, and find a positive association between self-reported political affiliation and regional pathogen stress for older (>40 years) but not younger individuals. It is concluded that the influence of environmental pathogen stress on ideology may have reduced over time.

Individual differences in political ideology have been ascribed to, for example, personality (Bakker, 2022; Gerber et al., 2011; Mondak, 2010), moral foundations (Haidt & Graham, 2007), genetic factors (Dawes et al., 2014), and family upbringing (Jennings & Niemi, 1968). However, these and other proposals often fail to specify *why* the relevant individual differences exist in the first place—what is their adaptive function? Many researchers have, therefore, examined how regional and temporal variations in ecological pressures influence and shape patterns of socio-political attitudes. The resulting studies have themselves produced mixed results. The central aim of the present article is to explore the possibility that the widely studied association between one specific ecological factor—parasite stress—and ideology may have reduced over time due to changes in the environment, leading to associations being weaker in younger people and in the present day.

A key ecological hypothesis is that the avoidance of infection-related death and disability has been a dominant evolutionary driving force throughout human history. According to parasite stress theory (e.g., Fincher & Thornhill, 2012; Schaller & Duncan, 2007; Thornhill & Fincher, 2014) different personalities and attitudes reflect adaptive responses of a behavioral immune system that seeks to manage the risks of infection posed by the environment (Kramer & Bressan, 2021; Schaller et al., 2015; Schaller & Park, 2011). It has been suggested that conservative ideology may reflect one such adaptation. There are, broadly, three approaches to addressing this question, and these concern (a) the relationship between disgust sensitivity and conservatism, (b) physiological reaction to disgust-inducing stimuli, and (c) the relationship between conservative ideology and environmental levels of infection threat (the topic of this article).

First, disgust sensitivity is a key defense strategy against contamination that may be passed on via contact with other members of the same species (Oaten et al., 2009). Some evidence on disgust sensitivity appears consistent with the hypothesis that aspects of conservative ideology, particularly social conservatism, may reflect an adaptive response to the need to avoid infection. Individual measures of disgust sensitivity, particularly contamination disgust, are associated with political conservatism and voting (Aaroe et al., 2020; Brenner & Inbar, 2015; Inbar et al., 2009, 2012; O’Shea et al., 2022) (but see Tybur et al., 2010, 2015). Evidence regarding the particular dimensions of disgust or infectability concern that are relevant is, however, mixed (Billingsley et al., 2018; O’Shea et al., 2022) and may depend on the length of the relevant questionnaires (Fournier et al., 2021) and the particular stimuli that are used to elicit disgust (Elad-Strenger et al., 2020). Disgust sensitivity has also been associated with specific aspects of conservatism such as traditionalism and opposition to immigration (Aaroe et al., 2017; Brenner & Inbar, 2015; Clifford et al., 2023; Murray et al., 2013; Terrizzi et al., 2013; Tybur et al., 2016). Here again, however, the strengths of observed associations may be small (Tybur et al., 2016) or variable (Terrizzi et al., 2013).

Second, physiological responsiveness to disgust and threat has been argued to be associated with ideological preferences (Oxley et al., 2008; Smith et al., 2011). However, there have been several reported failures to find differential physiological responses to disgust in people with different political preferences (Bakker et al., 2020; Fournier et al., 2020; Osmundsen et al., 2022; Smith & Warren, 2020). Moreover, Shook and Oosterhoff (2020) found no evidence that differences in political ideology were produced by experimental manipulations of pathogen stress.

A third and more direct approach involves examining links between conservative ideology and measures of parasite stress within the local environment (O’Shea et al., 2022; Thornhill & Fincher, 2014). Changes in the ecological environment have been associated with individualistic attitudes in the United States and have been linked to changes in attitudes to gender inequality (Grossmann & Varnum, 2015; Varnum & Grossmann, 2016), historical levels of pathogen prevalence predict support of those underlying moral values that are associated with conservative attitudes (van Leeuwen et al., 2012), and ideology and partisanship are predicted by parasite stress across U.S. states (O’Shea et al., 2022). Studies linking environmental levels of infection to social and economic attitudes have, however, been criticized on a number of grounds (e.g., Bromham et al., 2018; Hackman & Hruschka, 2013b; Pollet, 2014; Pollet et al., 2014). Some of these criticisms relate to methodological issues (the ecological fallacy, cross-cultural validity of constructs, etc.) and we address these as far as possible with our methodology (see below). Others concern the mechanisms responsible for the putative association between environmental parasite stress and conservative behavior, and suggest that such an association may not directly reflect pathogen infection avoidance but instead may be due to sexual disgust (e.g., Billingsley et al., 2018; Tybur et al., 2015) and/or the adoption of fast life history strategies (Hackman & Hruschka, 2013b). We return to these accounts in our general discussion, as they provide potential mechanisms that could explain the empirical result which is at the core of the present article. The primary focus of the present article is on how any influence of pathogen stress on conservative attitudes changes over time. At this point, therefore, we can remain somewhat agnostic about the underlying mechanism.

Despite a strong theoretical rationale for linking behavioral immune system to political ideology, relatively few studies have examined the relation between actual voting behavior, or vote-based measures of ideology, and objective measures of parasite stress. Zmigrod et al. (2021) found links between pathogen stress levels and authoritarian attitudes across both countries and U.S. states, along with associations with conservative voting in the 2016 U.S. Presidential election (see also Brenner & Inbar, 2015; Inbar et al., 2012; O’Shea et al., 2022). Recent outbreaks of infectious disease allow the influence of disease prevalence on aspects of sociality to be tested in a different way. Do temporary but salient increases in human-to-human transmitted illnesses increase social conservatism? The evidence for such an effect is mixed at best. Using Senate election data from 34 U.S. states, Beall et al. (2016) found that salience of Ebola (measured as frequency of internet searches for the diseases) correlated positively with Republican voting intentions. However, these results have been criticized on methodological grounds (see Schaller et al., 2017; Tiokhin & Hruschka, 2017). Other results are limited to survey responses and produce conflicting results. Kim et al. (2016) reported a significant correlation between fear of Ebola and xenophobic tendencies based on a nationally representative sample of 1,000 Americans, while in a survey of six European countries (with 105 unique regions) Wamsler et al. (2023) found that exposure to the pandemic is positively associated with stronger ethnic national identities. In contrast, however, Eder et al. (2021) found no evidence of perceived or objective COVID threat on ethnocentrism.

In a recent review of this literature, Ackerman et al. (2021) questioned the plausibility of the idea that responses to the pandemic will reflect reactivity of the behavioral immune system. The authors observe that xenophobic responses to pathogen prevalence would be of little value in the modern world, where group identity is a weak marker of infection risk. Although it is possible that the behavioral immune system continues to be activated by the elevated presence of pathogen cues, it is also true that conservatives (e.g., Republicans) tend to be less concerned with the recent COVID pandemic.

Taking the data as a whole, inconsistencies in existing results and alternative interpretation of the role the behavioral immune system might play in the modern world suggest caution against overinterpreting variations in conservative attitudes as directly reflecting behavioral strategies for avoiding sources of pathogens. To shed further light on this issue, and explore possible reasons for weak or ambiguous results in much of the existing literature, here we examine whether the relationship between conservative ideology and infection prevalence may have changed over time. It is possible that, as has been shown for personality (Mullett et al., 2020), only older individuals or those born in a particular cohort will show strong associations between ideology and infection prevalence. To motivate this approach, consider three ways in which a behavioral immune system might work. The first possibility is that the sensitivity of the system is not responsive to changing levels of infection in the environment, or at least not sensitive in a way observable over a timescale of mere decades. We refer to this as the “prevalence-independent model.” A second possibility—a “prevalence-dependent model”—is that the system is sensitive to changes in prevailing infection levels, so that whenever levels are high it registers the increased threat level and responds by initiating generally conservative and infection-avoidant patterns of behavior, while at the same time becoming more responsive to (and repelled by) infection-relevant stimuli of any kind. A third possibility—the “early-set model”—is that the general sensitivity of the system is either inherited (“early-set-inherited” model, reflecting direct selection pressure) or becomes fixed during childhood. If the system is set during childhood, the setting could reflect either prevailing levels of infection at the time of childhood (“early-set-prevailing” model), or could be influenced by parental and/or grandparental transmission of infection-relevant norms (“early-set-parental” model).

A simple prevalence-independent model predicts no change in the infection-ideology relationship over time, whereas a prevalence-dependent model predicts that the infection-ideology relationship will weaken over time as levels of infection reduce. Early-set models also predict that the infection-ideology relationship will get weaker over time, following reducing infection levels. However, early-set models lead to the additional prediction that currently older individuals, who were born (or whose parents were born) at a time when infection levels were generally higher, will show a stronger relationship between local levels of infection and ideology.^1^ We assume that this stronger relationship will apply both for U.S. citizens who live in their birth states (the majority: Long, 1988; Molloy et al., 2011) and the substantial minority who do not. This is because the infection-responsiveness of people who have moved to a new region will, according to early-set models, depend on the environment they experienced in their early years, just as will the infection-responsiveness of people who have not moved. Such a relationship could also be predicted by a prevalence-dependent model supplemented by a lifespan effect (e.g., the immune systems of older people are weaker, so older people should be more sensitive to infection-related stimuli).

In the light of these considerations regarding possible changes in the infection-ideology relationship over time or relating to participant age, a major limitation of previous research is that the majority of studies have used young participants. For example, the metareview of the relationship between disgust sensitivity and various measures of social conservatism by Terrizzi et al. (2013) includes 24 studies. For 21 of these, the mean age could reasonably be estimated;^2^ the mean of the average participant ages in these studies was 22.8 (*SD* = 5.2). Although some more recent studies have used Amazon Turk participants, where mean ages are typically around 36 years, we are not aware of any systematic relevant study of effects of age. Some suggestion that age may be relevant is provided by an analysis of data reported in Tybur et al. (2016) of the relation between disgust sensitivity and traditionalism in 30 nations. Tybur et al. report the mean age of the participants in each of their country-specific samples, along with the associated correlation between traditionalism and disgust sensitivity. We found a significant positive correlation between mean ages and the associated correlations, *r*(28) = .47; *p* = .009, for the raw correlations; *r*(28) = .43; *p* = .017, for the correlations attenuated for unreliability. Although other factors may be relevant, this correlation is at least consistent with the suggestion that the relationship between conservative ideology and disease-relevant concerns may be stronger in older individuals.

Young people, particularly in the developed world (in which most studies have been undertaken) have both grown up in, and inhabit, a world in which levels of parasite stress are very low by evolutionary standards. Figure 1A shows the decrease in total mortality in the United States due to major infectious diseases (TB, malaria, typhoid, whooping cough, measles, and polio, i.e., excluding sexually transmitted diseases [STDs]; see Methods for data source). It is evident that infection-related mortality fell, by about 1980, to a level that is exceptionally low by historical standards. In recent decades, levels of infectious disease may therefore have become a weaker predictor of cognitive style and social attitudes such as conservatism due to the greatly reduced fitness-relevance of infection relative to other influences. Here, we, therefore, hypothesize that clearer understanding of any ideology/pathogen stress relationship may be obtained by (a) examining whether the relationship between parasite stress and people’s ideology has changed over time as levels of infections have reduced, and (b) testing whether associations are stronger in people who are currently older, as they or their parents will have grown up in times when levels of infectious disease were higher than in the present day (Mullett et al., 2020).

**Figure 1.**
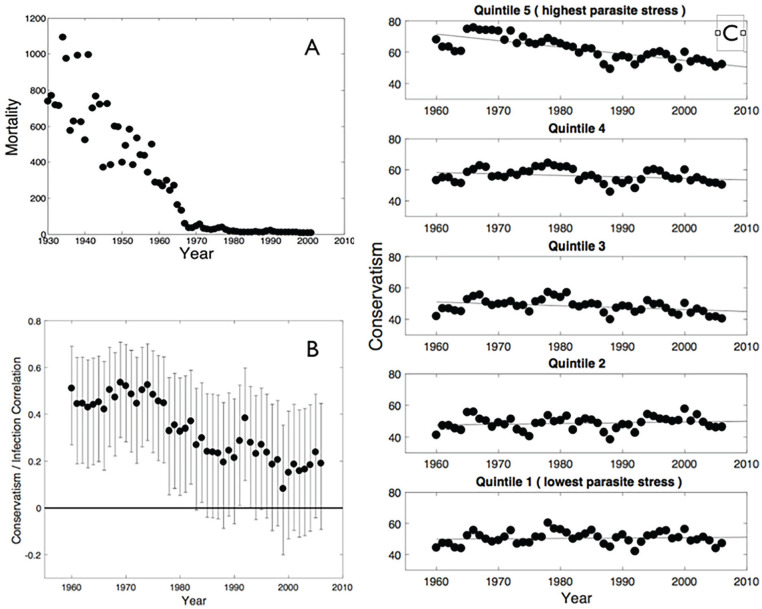
(A) Infection-Related Mortality Over Time (Age Adjusted Per 100,000) in the *United States* (Excluding Deaths Due to STDs). (B) Correlations Between Ideology and Our Measure of Parasite Stress (Averaged Over Years 1993 to 2007) in U.S. States Over Time. *Note.* The horizontal line corresponds to a correlation coefficient of 0; error bars represent 95% confidence intervals. C: State ideology over time for states in the different quintiles for parasite stress. Zero is “most liberal”; 100 is “most conservative” on the vertical axis. STD = sexually transmitted diseases.

Specifically, we hypothesize that as parasite stress in the environment reduces (as infection-related mortality decreases over time) its importance as an evolutionary or socio-cultural driver will also reduce, allowing other drivers to become more prominent in their effects. We, therefore, predict that there will be a reduction in its effect on ideology. We further hypothesize that any relationship between environmental pathogen stress and ideology will reflect early-life environment, and hence that the infection–ideology association will be weaker or absent in individuals who are, in the present day, younger rather than older.

There are limited data available to test this hypothesis, particularly data at the individual level that are necessary if analyses are to avoid the ecological fallacy (Pollet et al., 2014). We, therefore, adopt two converging methodologies. First, in Study 1, we conduct a cross-sectional analysis of state-level ideology in the United States and state-level pathogen stress. More specifically, we correlate a state-level measure of parasite stress (based on data collected between 1993 and 2007 and averaged over those years) with state-level measures of ideology collected for each year from 1960 until 2006. The measure of ideology is calculated by estimating the ideological position of members of congress and challengers for each congressional district using interest group ratings, then using voting records as a weighting factor (Berry et al., 1998). Second, in Study 2, we used individual-level data from more than 44,000 Facebook users to examine the relationship between age, self-reported ideological position, and state-level parasite stress.

## Study 1: State-Level Correlations of Parasite Stress and Ideology

### Data and Method

All information needed to reproduce the analysis is available at https://osf.io/nqj6e/. The data on state-level ideology are publicly available at https://dataverse.harvard.edu. The measures of parasite stress are based on data publicly available data at www.cdc.gov, and the derived measures are available as supplementary material to Fincher and Thornhill (2012) (for overall parasite stress) and in Thornhill and Fincher (2014) (for zoonotic and non-zoonotic measures). Corrected versions of the latter are available at https://core.ac.uk/display/149745841. The additional state-level measures of parasite stress based on mortality from various infectious diseases were obtained from the CDC WONDER Online Database (wonder.cdc.gov/DataSets.html) using that dataset’s classification of infectious diseases. The measure took the state-wise median infection-related mortality rates for the years 1968 to 1978 (code IDC-8).

All exclusions and manipulation measures are reported. Studies were not preregistered. Sample size was fixed ahead of time by data availability; the sample size required to detect a correlation of at least 0.5 with 80% power using a two-sided 5%-level test is 30 observations (here, observations are U.S. states).

#### Measures of State-Level Ideology

Our index of citizen state-level ideology was taken from Berry et al. (1998), who report a measure from 1960 to 1992 (updated measure through 2006 available from dataverse.harvard.edu). The measure assigns a score to each state for each year (where 0 is most conservative, 100 is most liberal; we reversed the coding for consistency between studies, so we use higher numbers to mean “more conservative”). The measure is calculated by estimating the ideological position of members of Congress and challengers for each congressional district using interest group ratings, then using voting records as a weighting factor. Measures for years when there are no elections are imputed (Berry et al., 1998). The Berry et al. measure is not without limitations (Shor & McCarty, 2011). As Shor and McCarty note, it provides no information about within-state heterogeneity and assumes that delegations to Congress reflect the same preferences as within-state delegations. Nevertheless, it is at present the best single unidimensional state-level measure of state-level ideology that is available over a long enough time period for the present study. It is assumed that an individual’s ideological position can be well captured by their position along a single dimension ranging from the political left (“liberal” in United States) to the political right. Although the use of a single dimension can represent an oversimplification at the individual level, when scaling techniques are used to position politicians in the light of their voting patterns little additional variance is accounted for by assuming a second dimension to the underlying ideological space (McCarty et al., 2006). At the level of individuals, social and economic conservatism appear to be different constructs (Everett, 2013), and the parasite stress hypothesis predicts that social, rather than economic, conservatism will be associated with infection prevalence (Terrizzi et al., 2013). Although voting patterns cannot distinguish between social and economic conservatism, the existence of a distinction can only add noise and hence act against our hypothesis.^3^

#### Measures of Parasite Stress

Our primary analysis examined the relationship between parasite stress and state-level ideology at the level of U.S. states (*N* = 50; Washington DC was excluded). The terms parasite and pathogen are here used to refer to any disease-causing infectious agents (e.g., viruses, bacteria, and helminths) however transmitted (e.g., by insect, air, water, food, direct contact). As our first index of parasite stress, we adopted the measure developed by Fincher and Thornhill (2012). This measure counts all infectious diseases reported by the U.S. Centers for Disease Control (CDC) for the years 1993 to 2007 for each state, divides the counts by state population, and transforms the result into a z-score (Fincher & Thornhill, 2012). There are insufficient data to enable separate measures of parasite stress for different years or other time periods; our analysis assumes that *relative* parasite stress in different U.S. states remains stable over time. Our approach is conservative in that the parasite stress measure was calculated based upon recent infection levels, but our hypothesis predicts an effect upon voting patterns only in earlier time periods (i.e., those for which the parasite stress measure that we used may be less reliable or noisier).

We also report analysis using alternative measures of parasite stress to test the robustness of our results. Following Fincher and Thornhill (2012), we distinguish between zoonotic and non-zoonotic parasitic stress. Zoonotic infectious diseases include infections that humans can only acquire from non-human species (livestock and wildlife); non-zoonotic infectious diseases are those that can be transmitted between humans, although humans may acquire them from non-human species. According to the parasite stress hypothesis, and assuming sufficient selectivity in the relevant processes, it is only the latter that should be related to forms of human interaction (because avoidance of unfamiliar humans is only adaptive in the context of non-zoonotic infections).

### Results

**Figure A1.**
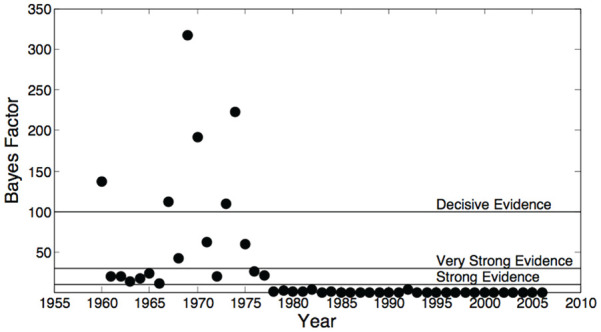
Bayes Factors for the Correlations Reported in Main Text Figure 1, Panel B.

We report descriptive statistics (zero-order correlations) between our measure of parasite stress and the index of state-level ideology. Tests are two-sided. Figure 1B shows the zero-order correlations between state-level parasite stress and state-level ideology for each available year. Although the correlations are not independent of each other, and hence the confidence intervals should not be over-interpreted, there is a clear pattern of strong positive correlations between parasite stress and conservatism—that is, high levels of infection are associated with more conservative ideology, as predicted. The correlations appear to get weaker in magnitude over time, and if considered in isolation would have failed to reach conventional significance from the early 1980s, although we note that, with an *N* of 50, this first study has limited power to detect small correlations. We calculated Bayes factors for the correlations between parasite stress and citizen ideology shown in main text Figure 1B (Wetzels & Wagenmakers, 2012). The resulting Bayes factors (see Appendix) are shown in Figure A1, where (although again noting the non-independence of years) it can be seen that there is at least “strong” evidence for an association in years 1960 through 1977.

Figure 1C shows state-level ideology over time for U.S. states separated into quintiles by overall level of parasitic stress. It can be seen that the high-parasite stress states have become more liberal since 1960, with little or no corresponding change in the lower-parasite stress states.

**Figure 2.**
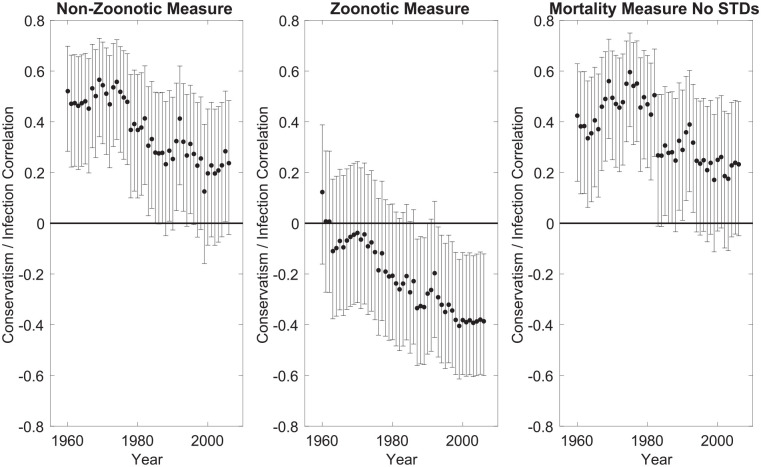
Correlations Between Ideology and Three Different Measures of Parasite Stress. *Note*. The horizontal line corresponds to a correlation coefficient of 0; error bars represent 95% confidence intervals.

According to the parasite stress theory, conservative ideology should be driven by the threat of infection from other humans (i.e., non-zoonotic infections) and not affected by threat of infection from different species (i.e., zoonotic infections). As Figure 2 (Panel 1) shows, there is a positive association between non-zoonotic parasite stress and conservatism, but this association reduces over time. The effects of zoonotic parasitic stress, in contrast, are either non-significant or opposite in sign (Figure 2, Panel 2). These results are consistent with the hypotheses that (a) the effects of parasite stress reduce over time and (b) the positive relationship between parasitic stress and conservatism is specific to measures of non-zoonotic parasite stress.

To examine the robustness of the observed associations, we repeated our analysis using an additional measure of the parasite stress burden based on mortality. We used mortality data from CDC for the years 1968 to 1978 (ICD-8), excluding STDs, as they best cover the years for which we found strong associations between parasite stress and ideology in the previously-reported analyses and because there were many fewer infection-related deaths in subsequent years. The measure complements the Fincher and Thornhill measure of parasite stress used in Figure 1, as it uses mortality data alone; we excluded STD-related mortality from the measure to reduce confounds with life history strategies (see Zhang et al., 2015; and also General Discussion). The results are shown in Figure 2, Panel 3, where it can be seen that, as with the non-zoonotic measure, correlations between parasite stress and conservative ideology reduce over time. These results should however be treated with caution as the mortality-based measure relates to the earlier years of the examined period.^4^

The analyses reported above used state-level measures of ideology. Such measures do not allow us to relate individual measures of ideology to regional levels of parasite stress and the results are open to the ecological fallacy (i.e., making illegitimate inferences about individual-level relationships from group-level data). Moreover, we did not include time-varying or other controls that may underlie the observed associations because (a) the parasite stress measures, which do not vary over time, are too limited to enable comparison with the time-varying controls that would be necessary or to allow exploitation of the longitudinal element of the voting data, especially given the likely importance of autocorrelation effects, and (b) the analysis cannot distinguish between cohort effects and period effects (see below). The results of Study 1 are, therefore, best seen as consistent with the idea that conservative ideology reflects infection prevalence while at the same time being open to a number of alternative potential explanations.

In Study 2, we, therefore, examined the role of pathogen prevalence on individuals’ conservativism as a function of their age using a much larger dataset. This approach enables us to examine in a completely different way whether the effect of pathogen stress on ideology has reduced over time. In particular, the approach allows us to distinguish between period effects and cohort effects. If reducing parasite stress influences all age groups in a similar way (a period effect) we would expect no interaction between age and parasite stress as predictors of ideology. If, on the other hand, it is only people who were born at a time relating to when parasite stress levels were high (i.e., a cohort effect exists), we would expect parasite stress to be associated with ideology only, or more strongly, for people who are older at the time of data collection. Given the assumption that the behavioral immune system’s sensitivity reflects conditions in early life, we predict that older individuals, who grew up (or whose parents grew up) in a period when infection rates were substantially higher (and hence represented greater mortality risk) than they are now, are likely to exhibit a stronger relationship between their ideological views and prevailing levels of infections in the regions they inhabit.

## Study 2: Age-Specific Associations Between Ideology and Parasite Stress

### Data and Method

For this second study, measures of state-level median household income, income inequality (GINI), and total population were obtained from the 2012 American Community Survey available from the U.S. Census Bureau data repository (https://www.census.gov/). Sharing of the individual-level Facebook data would compromise participants’ privacy and constitute an IRB ethics violation. Details of the dataset and the policies under which it was obtained can be found at https://sites.google.com/michalkosinski.com/mypersonality.

#### Measures

The data were collected using the Facebook application myPersonality (Kosinski et al., 2015). This application was launched in 2007 and rapidly gained popularity. By the time it closed in 2012, over 3 million unique Facebook users had completed at least one service provided by the application. There was a range of measures available to individuals, but the most popular was the 20-question measure of the International Personality Item Pool (IPIP) which provided participants with an estimate of their scores on the Big 5 personality model (Goldberg et al., 2006). Participants received no payment and completed the survey to receive information and feedback regarding their personality which they could then share using Facebook’s social networking tools. After completing the survey, participants were asked if they would consent to their responses and Facebook profile information being used for research purposes. This profile information contains much of the information which would be collected during traditional psychology experiments, including gender, age, and current country and state of residence. In addition, users were able to enter “Political Views” in a free text field. It is this information that we use here to measure political ideology.

For this analysis, we restrict our dataset to myPersonality users from the United States. We used the free text response given in the “Political Views” section of individuals’ Facebook profiles to create an empirical measure of political ideology. The two-party system in the United States makes this simpler than in other countries. After restricting the dataset to include only participants who identified themselves as living within a U.S. state and who entered a response in the “Political Views” field, and excluding 548 participants under the age of 16, we were left with 44,298 respondents. The sample size required to detect an interaction effect of at least 0.05^5^ with 80% power is 3100 observations (here survey respondents). Political views were encoded as “conservative” for all individuals who responded “Republican,” “Conservative” or “Very Conservative” They were encoded as “liberal” for all those who entered “Democrat,” “Liberal” or “Very Liberal.” Of those who responded to the “Political Views” field, 72.3% could be categorized. Of these, 74.6% were liberal and 25.4% were conservative. Of those who were not categorized, the majority responded as “other” (36%) or “moderate” (31%), with smaller numbers responding “none” (12%), “libertarian” (8%), apathetic (6%) or “independent” (5%). All other responses occurred fewer than 100 times each. Political affiliation of each categorizable user was coded as 1 (conservative) and 0 (liberal).

The demographics of the reduced sample were representative of the Facebook population, with a gender bias of 62% females and a mean age of 27. In our analysis, we also controlled for state-level characteristics, including median household income, income inequality (GINI), and total population.

#### Results

A mixed model approach was used to estimate the relationship between parasite stress and ideology. State-level controls were included. The mixed model methodology allows us to enter individual-level and state-level variables, while controlling for random effects at the level of the state. A separate model is estimated for each measure of parasite stress, but using the same controls and fitting parameters for each. Ideology was encoded with conservative as 1 and liberal as 0. A positive beta coefficient, therefore, indicates increased likelihood of being conservative. The variables of interest were state-level measures of parasite stress, and the interaction of these measures with age. We used the zoonotic and non-zoonotic measures of parasite stress that were used in Study 1.^6^ We also used the mortality-based measure (excluding STDs) that we used in Study 1, but supplemented this measure with mortality statistics based on specific (mainly younger) age groups to aid theoretical interpretation.

For age, random intercepts and slopes were estimated for the level of the state, as was gender (Barr et al., 2013). In all models, we control the effects of income inequality, state population, urban-rural ratio, and median household income. Tests are two-sided.

We first used multi-level modeling to estimate the relationship between the level of parasite stress in each person’s state of residence and his or her ideological stance, while controlling for a range of state-level socio-economic characteristics. The regression results are shown in Table 1 for all three non-age-specific measures of parasite stress (zoonotic infections, non-zoonotic infections, and mortality). As expected, for the non-zoonotic and mortality-based measures of parasite stress, there is a positive interaction between age and parasite stress in the predicted direction such that the relation between parasite stress and conservative ideology became more positive in older participants. When the zoonotic measure of parasite stress is used, however, there is (again as predicted) no evidence of an interaction between age and parasite stress.

**Table 1.** Logistic Model Predicting Individuals’ Self-Reported Ideology From State-Level Parasite Stress (Three Measures).

	Non-zoonotic		Zoonotic		Mortality no STDs	
	Beta	95% CI	Beta	95% CI	Beta	95% CI
Intercept	14.840***	[9.400, 20.280]	13.060***	[5.980, 20.150]	14.400***	[9.460, 19.350]
Population	−0.040	[−0.160, 0.070]	0.030	[−0.120, 0.170]	0.010	[−0.090, 0.120]
GINI	−11.540***	[−17.790, −5.300]	−9.520*	[−17.690, −1.350]	−13.530***	[−19.430, −7.630]
Income	−1.530***	[−2.200, −0.870]	−1.470***	[−2.250, −0.680]	−1.430***	[−2.040, −0.830]
Urbanization	0.000	[−0.030, 0.020]	−0.010	[−0.040, 0.020]	0.000	[−0.020, 0.020]
Gender	−0.240***	[−0.290, −0.190]	−0.240***	[−0.290, −0.190]	−0.240***	[−0.290, −0.190]
Age	0.010***	[0.010, 0.010]	0.010***	[0.010, 0.010]	−0.010	[−0.020, 0.000]
Parasite stress	2.200	[−11.040, 15.440]	1.480	[−19.430, 22.390]	4.120	[−1.360, 9.600]
Parasite Stress × Age	0.610***	[0.320, 0.910]	0.080	[−0.430, 0.600]	0.250***	[0.120, 0.380]
BIC	61,569.39		61,586.14		61,559.97492	
LL	−30,757.3		−30,765.7		−30,752.6033	

*Note.* CI = confidence interval; STD = sexually transmitted diseases; BIC = Bayesian information criterion; LL = lower limit.

* *p* < .05. **p < .01. ***p < .001.

**Figure 3.**
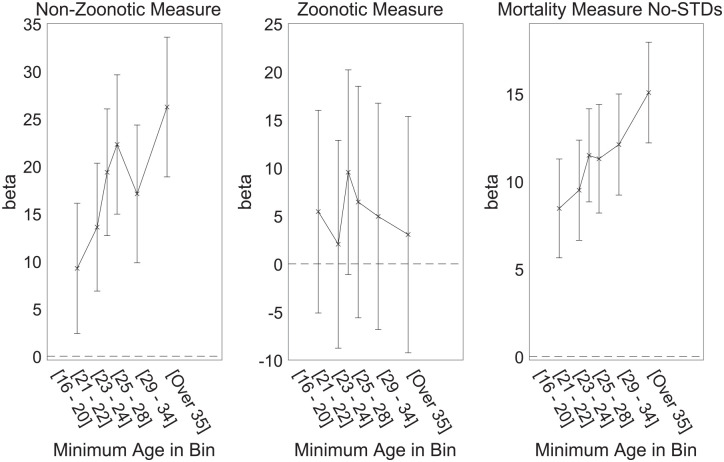
Interaction Between Participant Age and Effect of State-Level Parasite Stress on Ideology for Three Measures of Parasite Stress. *Note.* Means and 95% confidence intervals are shown. Values/position on the x-axis are defined using the lowest age in each bin.

To enable visualization of the effects of age and parasite stress, we repeated the analysis but with age and its interaction term removed. Instead, we split the data by age into six bins, each with equal number of participants. The model was then estimated separately for each bin. The resulting beta coefficients are shown plotted against age in Figure 3 separately for each measure of parasite stress. The effect of parasite stress is strongest in those near the age of 40 and older, and smallest among those in their late 20s and younger.

These results are, therefore, consistent with the idea that conservatism at the individual level is positively related only to measures of non-zoonotic parasite stress.

One possible explanation of the relation between environmental levels of parasite stress and social conservatism is that high levels of infection-related childhood mortality lead to behavior that maximizes the chances of survival at least to reproduction age. For this explanation to be tenable, conservatism must be predictable by measures of how parasite stress influences the survival of children and young adults. If parasite stress increases mortality primarily in older age groups rather than influencing the fitness of people of reproduction age, it is unlikely that any effect of parasite stress on ideology would be reflecting direct reproductive pressure.^7^ We, therefore, conducted three new analyses that differed in the mortality-based parasite measure that was used. The first analysis was based on mortality rates for people aged < 16, the second used mortality rates for people aged < 36, and the third used mortality rates for people aged >35. The aim here is to exclude the possibility that the infection–ideology link reflects only effects of parasite stress on older individuals (here, mortality rates for people aged >35).

**Figure 4.**
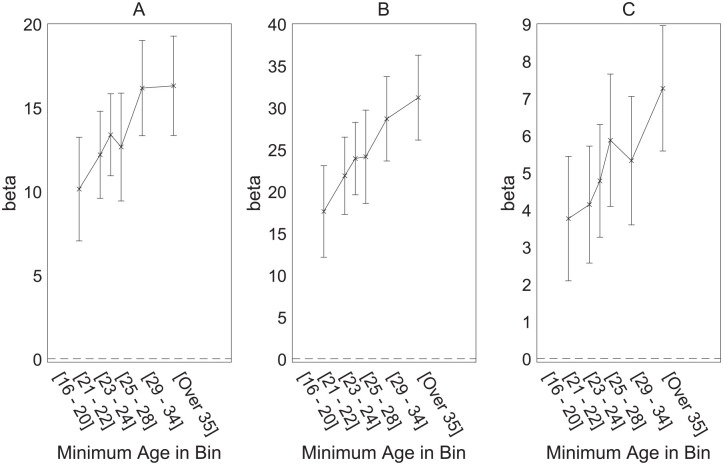
Interaction Between Participant Age and Effect of Three Different Age-Specific Mortality-Based Measures of Parasite Stress. (A) Parasite Stress Measure Based on Mortality Rate for 0- *to* 15-Year-Olds. (B) Parasite Stress Measure Based on Mortality Rate for 0- to 35-Year-Olds. (C) Parasite Stress Measure Based on Mortality Rate for >35-Year-Olds; Means and 95% Confidence Intervals Are Shown. *Note.* Values/position on the x-axis are defined using the lowest age in each bin.

The results of these regressions are shown in Table 2, and the critical interactions between age and parasite stress are shown in Figure 4. The three separate panels each show results for all participants but differ in the age range on which the parasite stress measure is based. It can be seen that the key interaction is seen with all three age-specific mortality-based measures of parasite stress. These results appear consistent with the possibility that the relation between parasite stress and ideology reflects reproductive potential; we defer further consideration to the “General Discussion” section.

**Table 2.** Model Predicting Individuals’ Self-Reported Ideology From Three State-Level Age-Specific Mortality-Based Measures of Parasite Stress.

	0- to 15-year-olds		0- to 35-year-olds		>35-year-olds	
	Beta	95% CI	Beta	95% CI	Beta	95% CI
Intercept	9.240***	[4.390, 14.090]	9.630***	[4.910, 14.340]	15.130***	[9.780, 20.480]
Population	0.020	[−0.080, 0.110]	0.020	[−0.080, 0.110]	0.010	[−0.100, 0.120]
GINI	−12.840***	[−18.150, −7.540]	−13.650***	[−18.940, −8.370]	−12.150***	[−18.330, −5.980]
Income	−0.660*	[−1.310, −0.010]	−0.670*	[−1.300, −0.030]	−1.630***	[−2.270, −0.990]
Urbanization	−0.010	[−0.030, 0.010]	−0.010	[−0.030, 0.010]	0.000	[−0.030, 0.020]
Gender	−0.240***	[−0.290, −0.190]	−0.240***	[−0.290, −0.190]	−0.240***	[−0.290, −0.190]
Age	−0.010	[−0.020, 0.000]	−0.010*	[−0.020, 0.000]	−0.010	[−0.020, 0.000]
Parasite stress	5.730*	[0.250, 11.210]	10.430*	[0.850, 20.000]	1.600	[−1.610, 4.800]
Parasite Stress × Age	0.280***	[0.160, 0.400]	0.520***	[0.320, 0.730]	0.120**	[0.040, 0.200]
BIC	61,548.14		61,543.91		61,569.69	
LL	−30,746.7		−30,744.6		−30,757.5	

*Note.* CI = confidence interval; BIC = Bayesian information criterion; LL = lower limit.

* *p* < .05. **p < .01. ***p < .001.

## General Discussion

We set out to explore environmental determinants of individual differences in ideology. We tested the hypothesis that an important component of political ideology lies in the environmental threats of contracting infectious diseases and that the relationship between ideology and infectious diseases will have become weaker over time due to the general reduction in infection-related mortality.

Our first study showed that higher regional (U.S. state-level) mortality due to human-transmitted diseases is correlated with more conservative voting patterns, but that this correlation has become smaller over time: The correlations in the years since about 1985 are only half as large as the correlations in the 1960s and 1970s, even when the measure of parasite stress was taken from later years (1993–2007). We interpret these results as consistent with the hypothesis that the link between conservative ideology and high infection threat has reduced. However, the first study only examined aggregated effects and hence could not establish a relationship at the individual level, and the interpretation is limited by possible omitted variable bias. Our second study examined individual-level ideology and found that the relationship between regional level of parasite stress and individual’s conservatism is stronger for currently older adults than for currently younger adults. These results were unchanged by inclusion of covariates for a range of state-level socio-economic characteristics. We note that, in Study 2, the state-level measure of parasite stress, as a measure of environmental characteristics rather than a measure of individual differences such as sensitivity to disgust, was necessarily aggregated spatially and was also averaged over years.

Even though the two studies involve different methodologies and different datasets, they both identify time-related changes in the association between parasite stress and ideology. What specific mechanisms might underpin these changes?

We first consider whether the overall pattern of results is more suggestive of a lifespan effect (older people show a stronger relationship between levels of infection and ideology simply because they are older and hence likely have weaker immune systems), a period effect (changes in parasite stress levels affect everybody, irrespective of age or date of birth) or a cohort effect (only people born at a particular time will show a relationship between ideology and levels of parasite stress). Study 2 found a clear tendency for older people to show a stronger association between ideology and parasite stress, suggesting that our results cannot be interpreted as a period effect. However, older people might show stronger associations because they are older (a lifespan effect) or because they were born at a different time (a cohort effect). Although the Study 2 results cannot distinguish between these two interpretations, the results of Study 1 might appear to provide partial evidence against a lifespan effect and consistent with a cohort effect. In the United States, the proportion of older individuals in the population has increased over time. Thus, if age per se is the relevant factor, the ideology-infection link in the population as a whole might be expected to grow over time, rather than (as is observed in Study 1) reduced. However, the aging of a given cohort is confounded with the reducing parasite stress over the same time period, and it is impossible to tell whether the latter effects might be stronger than the former. Nonetheless, we interpret the pattern of data taken as a whole as likely reflecting a cohort effect rather than a period or a lifespan effect. In the introduction, we distinguished between prevalence-independent, prevalence-dependent, and early-set models of how a behavioral immune system might work. Our findings that infection-ideology associations change over time are inconsistent with a prevalence-independent model, and the results of Study 2 (older adults show stronger associations between ideology and prevailing levels of parasite stress) appear more easily explained by an early-set model than by a prevalence-dependent account.

The relationship between cohorts’ birthdates and reducing parasite stress can constrain theoretical interpretations further. An adult born in 1970 would have been aged between 37 and 42 at the time they completed the Facebook personality questionnaire, and it is this age group that showed the strongest relationship between ideology and parasite stress (see Figure 3). However, an adult born in 1990 would have been aged between 17 and 22 at the time of data collection, and even this group showed a significant (although much smaller) ideology-infection link (Figure 3). As Figure 1 shows, infection-related mortality had already reduced to very low (by historical standards) levels by 1970. Thus, even the oldest group in our study was born into a relatively low-parasite stress environment, suggesting that the environment their parents or grandparents were born into is more relevant. The parents of our youngest (oldest) participants would have been born around 1965 (1945), when parasite stress levels were much higher than they were at the time the participants themselves were born. Parental transmission of infection-related concern is one mechanism that fits the time-lines seen in our data, although direct selection effects cannot be ruled out (i.e., people born in 1945 may have been more likely to survive to reproduction age if they had highly active behavioral immune systems, and this characteristic may have been inherited by their children).

A further issue concerns the mechanisms that underpin the association when it does occur. First, our results point to the importance of human social interactions in explaining our results, because we find infection–ideology associations only when the infection measure concerns diseases that can be acquired from other humans. We follow numerous other researchers in assuming that important aspects of social conservatism can, at least in part, be viewed as an adaptive response to the threat of infection. Example mechanisms can be illustrated computationally: Using a simple agent-based model of social group formation, it has been shown that social groups with more local (rather than long-distance) cooperative relationships form when the simulated infection risk of cooperating with more distant others increases (Brown et al., 2016). This effect captures the idea that there is a trade-off between the benefits to an individual of cooperating with as many others as possible and the possible risk of being infected by a person who may either show signs of infection or be a member of a “foreign” group with a possibly higher risk of harboring diseases that may compromise an immune system that has developed within a particular region-specific disease pattern. Consistent with this general interpretation, Mullett et al. (2020) found that older people living in regions with high parasite stress levels were particularly low in the personality trait of openness (which is itself closely linked to socially liberal political attitudes). One possibility, therefore, is that the increasing conservatism we have observed in individuals who grew up (or whose parents grew up) in regions of high parasite stress may in part reflect an infection-avoidance strategy.

As noted in the introduction, however, some of criticisms of the putative association between environmental parasite stress and conservative ideology suggest that such an association may not directly reflect pathogen infection avoidance. One such criticism concerns the potential role of reproductive strategies in shaping conservative attitudes. One possibility is that pathogen prevalence influences sexual disgust, which in turn motivates adoption of more conservative sexual behaviors and attitudes (e.g., concerning the number of sexual partners, or age of first sexual contact). Endorsement of socially conservative policies might, therefore, reflect more conservative attitudes toward sex and reproduction (Billingsley et al., 2018; Tybur et al., 2015; Tybur & Lieberman, 2016). An alternative, but related, explanation for the cross-sectional relation between measures of infection-related mortality (parasite stress) and a variety of social behaviors is life history theory. According to this explanation, environmental risks shape reproductive strategies. When risks are high, “faster” strategies are more prevalent, promoting early sexual maturation, reducing parental investment, and increasing the number of offspring. The fast-life history approach suggests that disease prevalence is an outcome of reproductive strategies, rather than a cause (see Billingsley et al., 2018; Sinn & Hayes, 2018). Consistent with this alternative account, Hackman and Hruschka noted that most causes of mortality in the parasite stress index are caused by STDs, which are a proxy for fast life history (Hackman & Hruschka, 2013a, 2013b). In their re-analysis of state-wise differences of homicides, religiosity, and strength of family ties, Hackman and Hruschka show that accounting for fast life history removes the effect of parasite stress (see also Zhang et al., 2015). Specifically, they show that the effect of parasite stress disappears when controlling for number of teenage pregnancies (a proxy of fast life strategies) and that the effects of parasite stress do not hold when outcome data are disaggregated by race.

Our data do not enable us to distinguish between different accounts of the infection-ideology association with confidence, although we note that our effects are found even with a measure of parasite stress that excludes STDs. Indeed, we used that measure specifically because of the issues raised by Hackman and Hruschka. We also note that attributing variability in social conservatism to different sexual reproductive strategies is another form of the claim that rates of infectious diseases are a significant source of ecological pressure; our contribution in the present article is the demonstration that this pressure changes over time.

### Limitations

Our research is subject to a number of limitations. As already noted, our data do not allow us to make specific claims about whether our association is mediated by sexual strategies (Billingsley et al., 2018; Tybur et al., 2015), related to avoidance of foreign norms (Karinen et al., 2019), or reflects concern with individual contacts rather than outgroup avoidance (O’Shea et al., 2022; Tybur et al., 2016; van Leeuwen & Petersen, 2018). We also note that the relationship between political beliefs and other types of threat is country-dependent (Brandt et al., 2021), and the same is true for parasite stress (Tybur et al., 2016; Zmigrod et al., 2021).

Also, as already noted, available data do not permit analysis with year-specific controls (see especially Study 1). A related point arises from the fact that, within a country, there is an inevitable confound between the age of particular cohorts and changing levels of parasite stress. Geographical mobility has also seen major age- and region-related changes over time (Plane, 1992). Only cross-country and cross-period analysis will enable resolution of such issues.

Although we include (in Study 2) controls for state-level urbanization, income inequality, and median income, it is possible that parasite stress is acting as a proxy for more general resource scarcity or other negative aspects of the environment. It is, for example, already well established that events occurring at particular times in life can have long-lasting effects on voter preferences (Ghitza et al., 2022) and that reaching adulthood when the general economic environment is poor (e.g., in recessions) can permanently influence narcissism (Bianchi, 2014), and job preferences (Cotofan et al., 2023), as well as attitudes to democracy (Krishnarajan et al., 2023) and immigrants (Cotofan et al., 2021).

Our data are merely correlational, and, although cross-sectional associations can (given appropriate priors) be interpreted as evidence for or against specific causal models (Brown et al., in press), this limitation should be kept in mind.

Finally, we note that socio-cultural conservatism is a complex and multi-dimensional construct, and one that is distinct from economic conservatism. Further research will be needed, as suitable data become available, to assess using large datasets the hypothesis that it is specifically social (rather than economic) conservatism that is associated with changing effects of parasite stress. A related point is that social norms change over time, and that the attitudes of an individual who identifies as socially conservative may have become more liberal. However, in our studies, the dependent measures are not measures of social conservatism that are in any way absolute. In other words, our claim is about changes in the relation between parasite stress and relative conservatism rather than the relationship between parasite stress and absolute conservatism.

### Summary and Conclusion

Our main novel result is that historically higher regional levels of parasite stress are associated with conservative ideology for older people but not for younger people. The 20th century saw a dramatic decline in mortality related to infectious diseases (Armstrong et al., 1999), and hence the relative importance of other factors in influencing political behavior may have increased (McCarty et al., 2006). Given the importance of infectious disease as an adaptive influence, with almost 50% of children failing to reach reproduction age for infection-related reasons until relatively recently in evolutionary history (Volk & Atkinson, 2013), it seems plausible that declining levels of parasite-related disease and mortality may be responsible for the infection-ideology association also reducing in recent decades.

## Appendix

We calculated Bayes factors for the correlations between parasite stress and citizen ideology shown in main text Figure 1B (Wetzels & Wagenmakers, 2012). As recommended by Wetzels and Wagenmakers, we assumed Jeffreys-Zellner-Siow priors (Liang et al., 2008) and, following normal practice, we interpreted Bayes factors of > 10, > 30, and > 100 as “strong,” “very strong,” and “decisive” amounts of evidence, respectively (Jeffreys, 1961). The resulting Bayes factors are shown in Figure A1, where (although again noting the non-independence of years) it can be seen that there is at least “strong” evidence for years 1960 through 1977.

## Notes

1. We note that this hypothesis excludes the possibility that correlations might be stronger in times of lower pathogen stress because more children survived to reproduction age and hence there is more population variation in the relevant quantities.

2. For example, if the participants were stated to be undergraduates or to have participated for course credit. In two cases only median age is given; we included these as if they were averages.

3. The only other dataset which (a) goes sufficiently back in time, and (b) has some measures that could potentially distinguish between social and economic conservatism is the American National Election Study, but the sample size is insufficient for present purposes.

4. The apparent discontinuities in Figure 2 are not systematic; we note that (a) the measures of parasite stress are not time-varying, and (b) that apparent discontinuities occur at different years for different parasite stress measures even though the ideology measures are the same. Even the largest change between successive years (between 1982 and 1983 with the mortality measure) is not statistically significant at the conventional level (*z* = 1.365; *p* = .086).

5. The effect size (.05) refers to how much the correlation between age and ideology changes when conditioned on parasite stress, and equivalently how much the correlation between parasite stress and ideology changes when conditioned on age (i.e., it is the coefficient on the interaction term in the regression equation). The power analysis was carried out using the R package InteractionPoweR (Baranger, 2021) and was based on a correlation between age and parasite stress of .01, a correlation between age and ideology of .05, and a correlation between parasite stress and ideology of .05.

6. An additional analysis found that use of the combined (zoonotic and non-zoonotic together) measure of parasite stress led to similar effects to those (reported) of the non-zoonotic measure, although the critical interaction between parasite stress and age did not reach conventional levels of significance after inclusion of an additional control variable that was requested by reviewers.

7. We are grateful to an anonymous referee and the editor for this point and for suggestions for additional analyses.
